# Preclinical transgenic and patient‐derived xenograft models recapitulate the radiological features of human adamantinomatous craniopharyngioma

**DOI:** 10.1111/bpa.12525

**Published:** 2017-05-17

**Authors:** Jessica K. R. Boult, John R. Apps, Annett Hölsken, J. Ciaran Hutchinson, Gabriela Carreno, Laura S. Danielson, Laura M. Smith, Tobias Bäuerle, Rolf Buslei, Michael Buchfelder, Alex K. Virasami, Alexander Koers, Owen J. Arthurs, Thomas S. Jacques, Louis Chesler, Juan Pedro Martinez‐Barbera, Simon P. Robinson

**Affiliations:** ^1^ Division of Radiotherapy and Imaging The Institute of Cancer Research London UK; ^2^ Developmental Biology and Cancer Programme, Birth Defects Research Centre, UCL Great Ormond Street Institute of Child Health University College London London UK; ^3^ Department of Neuropathology University Hospital Erlangen, Friedrich‐Alexander University Erlangen‐Nürnberg Erlangen Germany; ^4^ Histopathology Department Great Ormond Street Hospital for Children NHS Foundation Trust London UK; ^5^ Division of Clinical Sciences The Institute of Cancer Research London UK; ^6^ Institute of Radiology, Preclinical Imaging Platform Erlangen (PIPE) University Hospital Erlangen, Friedrich‐Alexander‐Universität Erlangen‐Nürnberg Erlangen Germany; ^7^ Institute of Pathology Sozialstiftung Bamberg Bamberg Germany; ^8^ Department of Neurosurgery University Hospital Erlangen, Friedrich‐Alexander University Erlangen‐Nürnberg Erlangen Germany

**Keywords:** adamantinomatous craniopharyngioma, genetically engineered mouse models, magnetic resonance imaging, microcomputed tomography, patient‐derived xenografts

## Abstract

To assess the clinical relevance of transgenic and patient‐derived xenograft models of adamantinomatous craniopharyngioma (ACP) using serial magnetic resonance imaging (MRI) and high resolution post‐mortem microcomputed tomography (μ‐CT), with correlation with histology and human ACP imaging. The growth patterns and radiological features of tumors arising in *Hesx1^Cre/+^;Ctnnb1^lox(ex3)/+^* transgenic mice, and of patient‐derived ACP xenografts implanted in the cerebral cortex, were monitored longitudinally *in vivo* with anatomical and functional MRI, and by *ex vivo* μ‐CT at study end. Pathological correlates with hematoxylin and eosin stained sections were investigated. Early enlargement and heterogeneity of *Hesx1^Cre/+^;Ctnnb1^lox(ex3)/+^* mouse pituitaries was evident at initial imaging at 8 weeks, which was followed by enlargement of a solid tumor, and development of cysts and hemorrhage. Tumors demonstrated MRI features that recapitulated those of human ACP, specifically, T_1_‐weighted signal enhancement in the solid tumor component following Gd‐DTPA administration, and in some animals, hyperintense cysts on FLAIR and T_1_‐weighted images. *Ex vivo* μ‐CT correlated with MRI findings and identified smaller cysts, which were confirmed by histology. Characteristic histological features, including wet keratin and calcification, were visible on μ‐CT and verified by histological sections of patient‐derived ACP xenografts. The *Hesx1^Cre/+^;Ctnnb1^lox(ex3)/+^* transgenic mouse model and cerebral patient‐derived ACP xenografts recapitulate a number of the key radiological features of the human disease and provide promising foundations for *in vivo* trials of novel therapeutics for the treatment of these tumors.

## INTRODUCTION

Adamantinomatous craniopharyngioma (ACP) is the most common tumor of the sellar region in childhood, accounting for approximately 1.2–4% of pediatric intracranial tumors. Peak incidence occurs at 5–9 years with a second peak in adults aged 45–60 years 20, 21. Although appearing histopathologically benign and classified by the World Health Organisation as grade I 17, ACP is often clinically aggressive, demonstrating invasion of the hypothalamus and visual pathways, and destruction of the pituitary. Current treatment strategies involve surgery, radiotherapy and cystic drainage. Although associated with good 5 year survival, patients frequently suffer severe long term morbidity, with pituitary and hypothalamic dysfunction, visual impairment and poor quality of life 20.

ACP is an epithelial lesion thought to arise from Rathke's pouch, the embryonic primordium of the anterior pituitary, and is characterized by the formation of a peripheral basal layer of palisading epithelium, loose aggregates of stellate cells, nodules of “wet keratin” (anuclear “ghost cells”), whorl‐like cell clusters and cysts containing high levels of protein, cholesterol and calcification 19. ACPs present as variably cystic/solid tumors in the intrasellar and/or suprasellar region by both magnetic resonance imaging (MRI) and computed tomography (CT) 10. Calcification, present in over 90% of ACPs, is detectable by CT, which is therefore widely used for differential diagnosis following initial identification by MRI 29.

Mutations in *CTNNB1*, which encodes β‐catenin, predicted to cause the over‐activation of the WNT pathway, have been identified in the majority of human ACP samples analyzed 7, 9, 15, 24. Unusually, nuclear‐cytoplasmic localization of activated β‐catenin is found in only a small proportion of tumor cells, either as small cell clusters, which broadly correlate with epithelial whorls, or otherwise dispersed in a minority of cells throughout the tumor 9, 11. We have recently described the 3D structure of human ACP using high resolution micro‐CT (µ‐CT) imaging 2, and corroborated the presence of clusters at the leading invasive edge of tissue invasion 8, 26.

Expression of oncogenic β‐catenin in the developing pituitary in *Hesx1^Cre/+^;Ctnnb1^lox(ex3)/+^* mice results in tumors resembling human ACP at the histological and molecular levels 11. Despite activation of β‐catenin in all pituitary cells in this model, nuclear accumulation is only observed in small clusters of cells, analogous to human clusters. Previous studies have shown that these mice have a median survival of approximately 11 weeks, with a remarkable variability, and at the time of death exhibit large cystic masses beneath the brain 11. To date, the kinetics of tumor development in this transgenic mouse model of ACP has not been studied.

A patient‐derived xenograft model of ACP has also been established through placement of uncultivated human tumor tissue into the cerebral hemisphere of immunodeficient mice 26. Lesions propagated in this manner show comparable tissue architecture and express similar immunohistochemical markers to the patient tumors, including whorl‐like cell clusters with nuclear β‐catenin accumulation and activated EGFR 26.

In this study we have further characterized tumors arising in the *Hesx1^Cre/+^;Ctnnb1^lox(ex3)/+^* transgenic mouse model of ACP *in vivo*, describing both their radiological features and patterns of growth through longitudinal multiparametric MRI imaging. Using μ‐CT we have assessed both these murine tumors, and cerebral patient‐derived ACP xenografts, *ex vivo* in 3D at high resolution. Comparison with clinical imaging and pathology demonstrated that both these models recapitulate the radiological features of human ACP.

## MATERIALS AND METHODS

### *Hesx1^Cre/+^;Ctnnb1^lo^^x(ex3)/+^* transgenic model of ACP

*Hesx1^Cre/+^;Ctnnb1^lox(ex3)/+^* mice have been described previously 11. All experiments were performed in accordance with the local Animal Welfare and Ethical Review Board, the UK Home Office Animals (Scientific Procedures) Act 1986, the United Kingdom National Cancer Research Institute guidelines for the welfare of animals in cancer research 30 and the ARRIVE (animal research: reporting *in vivo* experiments) guidelines 16. Eighteen *Hesx1^Cre/+^;Ctnnb1^lox(ex3)/+^* and four wildtype control mice were assessed in this study.

### Patient‐derived ACP xenograft model

A fresh surgical specimen from a 56 year old male patient with ACP was retrieved from the Department of Neurosurgery at the University Hospital Erlangen. Tumor tissue was verified and classified as previously and was found to bear a TCT(Ser)>TGT(Cys) mutation in codon 33 of *CTNNB1* but no mutation in exon 15 of *BRAF* 13, 26. A declaration of consent for the patient is available, approved by the local ethics committee of the Friedrich‐Alexander‐Universität Erlangen‐Nürnberg. Procedures were conducted in accordance to the Declaration of Helsinki.

All experiments performed were authorized by the local government (Regierung von Unterfranken, Germany, Ref.‐No. 54–2532.01–25/14) in accordance with the animal protection act. The surgical specimen was divided and implanted into the right cerebral hemisphere of four female NMRI‐Fox1^nu^/Fox1^nu^ mice (Janvier Labs, Le Genest‐Saint‐Isle, France). Anesthetized mice were secured in a stereotactic frame (Bilaney Consultants, Düsseldorf, Germany), a 5mm incision made in the scalp and a 1mm burr hole drilled 3mm lateral to the bregma. A piece of uncultivated tumor tissue (1–8 mm^3^) was inserted through the hole using a sterile cannula and the skin closed using a suture (ETHILON*II 4–0, Ethicon, Norderstedt, Germany). Analgesic (1 mg/g metamizole, Ratiopharm, Ulm, Germany) was added to the drinking water for 3 days following the procedure. Body weight and animal behavior were monitored daily 26.

### Magnetic resonance imaging

^1^H MRI of *Hesx1^Cre/+^;Ctnnb1^lox(ex3)/+^* mice was performed on a 7T horizontal bore microimaging system (Bruker, Ettlingen, Germany) using a 30 mm birdcage coil and 1 mm thick slices acquired over a 25 mm × 25 mm field of view (FOV). Imaging was performed fortnightly from approximately 8 weeks of age, increasing to weekly once tumor progression was established. Anesthesia was induced with either 3% isoflurane in 100% oxygen (1 L/min) and maintained with 1% isoflurane (for longitudinal screening/monitoring), or a 10 mL/kg intraperitoneal injection of fentanyl citrate (0.315 mg/mL) plus fluanisone [10 mg/mL (Hypnorm; Janssen Pharmaceutical Ltd. High Wycombe, UK)], midazolam [5 mg/mL (Hypnovel; Roche, Burgess Hill, UK)], and sterile water (1:1:2) (for functional MRI at study end). A lateral tail vein was cannulated with a 27G butterfly catheter (Venisystems, Hospira, Royal Leamington Spa, UK) if remote administration of Gd‐DTPA (Magnevist; Schering, Berlin, Germany) was required. Core body temperature was maintained by warm air blown through the magnet bore.

Magnetic field homogeneity was first optimized by shimming over the entire brain using an automated shimming routine (FASTMAP). Anatomical multi‐slice contiguous T_2_‐weighted RARE images (T_R_=4500 ms, T_E_eff = 36 ms, 8 averages, 256 × 256 matrix, pixel size = 98 µm × 98 µm) were then acquired to monitor tumor development and for subsequent volumetric analysis. Fluid attenuated inversion recovery (FLAIR; *T*_R_=18 000 ms, *T*_E_eff = 35 ms, *T*_I_ = 2100 ms, 4 averages, flip angle = 125°, 128 × 128 matrix) and T_1_‐weighted (*T*_R_ = 1300 ms, *T*_E_ = 7.5 ms, 4 averages, 256 × 256 matrix) images were also acquired. At study end, echo‐planar diffusion‐weighted imaging (EPI‐DWI) was used to determine the apparent diffusion coefficient (ADC) 5, and either T_1_‐weighted images acquired before and 1 minute after intravenous administration of 0.1 mmol/kg Gd‐DTPA (Magnevist, Schering), or dynamic contrast‐enhanced (DCE) MRI 4 was used to assess patent tumor vasculature.

MRI of nude mice bearing cerebral patient‐derived ACP xenografts was performed between 124 and 130 days after tumor implantation, at which time successful tumor engraftment had previously been confirmed histologically 26, under isoflurane anesthesia, on a 7T horizontal bore microimaging system (Bruker, Ettlingen, Germany) equipped with a dedicated mouse brain coil and an animal monitoring system. T_2_‐weighted images (*T*_R_ = 2370 ms, *T*_E_ = 41 ms, slice thickness = 0.7 mm, FOV = 24 mm × 38 mm, 210 × 320 matrix, pixel size = 119 µm × 119 µm) and T_1_‐weighted contrast‐enhanced images prior to and following intravenous administration of 0.1 mmol/kg gadobutrol (Gadovist, Bayer Vital, Leverkusen, Germany) (*T*_R_ = 500 ms, *T*_E_ = 9 ms, slice thickness = 0.7 mm, FOV = 24 mm × 28 mm, 448 × 512 matrix, pixel size = 55 µm × 55 µm) were acquired.

### MRI data analysis

Volumetric analysis was performed using segmentation from regions of interest (ROIs) drawn on T_2_‐weighted images. Parameter estimation was undertaken using a Bayesian maximum *a posteriori* algorithm, which took into account the Rician distribution of noise in magnitude MR data to provide unbiased parameter estimates 27, 28. Estimates of the ADC (×10^−6^ mm^2^ s^−1^) were determined from the EPI‐DWI data. DCE MRI data were analyzed by incorporating the Tofts and Kermode pharmacokinetic model, from which the volume transfer constant (*K* ^trans^, minute^−1^), the rate of flux of contrast agent into the extracellular extravascular space within a given volume, was calculated 6.

All data were fitted on a pixel‐by‐pixel basis using in‐house software (ImageView, developed in IDL, ITT Visual Information Systems, Boulder, CO, USA), and the median value of each parameter determined from a ROI that encompassed either the whole brain, whole lesion or a portion of the lesion.

### Micro computed tomography

Intact heads from *Hesx1^Cre/+^;Ctnnb1^lox(ex3)/+^* mice and wildtype control mice were fixed in 10% formalin for at least 48 h, and patient‐derived xenograft‐bearing mouse brains were fixed in 4% formalin, prior to iodination in Lugol's iodine (I^127^ concentration of 2.94 × 10^−4^mol/mL) for at least 72 h to improve tissue contrast. The heads were then rinsed with distilled water to remove excess iodine, blotted dry and secured within a low density plastic container covered with polymer film to prevent specimen dehydration. Images were acquired using a Nikon XTH 225 ST micro‐(µ)‐CT scanner, utilizing a molybdenum X‐ray source with anode voltages ranging between 70 and 100 kV and detector exposure times of 500–708 ms over 3141 projections 2, 14. Data were reconstructed using CTPro3D (Nikon Metrology, Tring) and post‐processed with VG Studio MAX software (Volume Graphics GmbH, Heidelberg, Germany).

### Histopathology

Formalin‐fixed *Hesx1^Cre/+^;Ctnnb1^lox(ex3)/+^* mouse tissue imaged by µ‐CT was decalcified in 1% formic formaldehyde for 48 h before embedding in paraffin blocks. A further 5 surface decalcifications of 2 h each were performed prior to 5 µm sections being cut in the axial or sagittal plane, matched to the imaging plane. Sections were then stained with hematoxylin and eosin (H&E).

The brains from patient‐derived xenograft‐bearing mice were formalin‐fixed and processed following µCT imaging, and H&E staining of 3 µm axial sections was performed as previously described 26.

Slides were scanned using either Nanozoomer (Hamamatsu Photonics, Welwyn Garden City) or Pannoramic MIDI (3D‐Histech, Sysmex Europe) and processed using CaseViewer 2.0 software (3D‐Histech, Budapest, Hungary).

### Statistics

Statistical and survival analysis was performed with GraphPad Prism 7 (GraphPad Software, La Jolla, CA, USA). The mean of median values for quantitative MRI parameters were reported and used for statistical analysis. Results are presented as the mean ± 1 standard error of the mean (SEM). Significance testing used Student's unpaired t‐test and Pearson correlation coefficient (one tailed) with a 5% confidence level.

## RESULTS

### *Hesx1^Cre/+^;Ctnnb1^lox(ex3)/+^* mice develop cystic‐solid tumors with a nonlinear growth pattern

**Figure 1:**
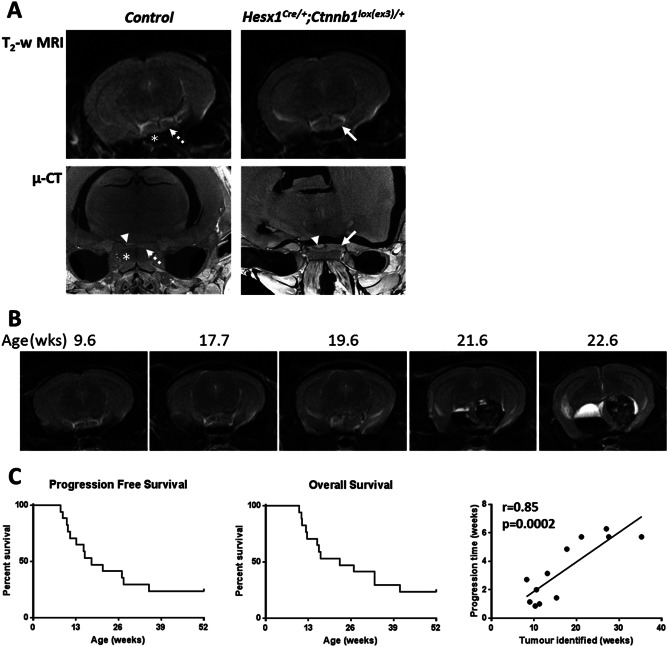
**A.** Axial *in vivo* T_2_‐weighted MRI (upper panel) and *ex vivo* micro (µ)‐CT (lower panel) images of the pituitary region of 8 week old control and *Hesx1^Cre/+^;Ctnnb1^l^°^x(ex3)/+^* mutant mice (each image acquired from a different mouse). Note the expansion and increased heterogeneity of the *Hesx1^Cre/+^;Ctnnb1^l^°^x(ex3)/+^* pituitaries (solid arrows) relative to the controls (dashed arrows). Arrowheads indicate the posterior lobe of pituitary also detectable in μ‐CT images and * denotes the sphenoid bone in control mice. MRI resolution 98 × 98×1000 µm; µ‐CT resolution approximately 9 µm isotropic. **B.** T_2_‐weighted MRI images of a *Hesx1^Cre/+^;Ctnnb1^l^°^x(ex3)/+^* mouse demonstrating the evolution of a tumor. In this mouse the first remarkable change was detected at 17.7 weeks, which was followed by rapid tumor progression including growth of the solid component, cyst formation and hemorrhage; the mouse was humanely killed at 22.6 weeks according to Home Office regulations. **C.** Progression‐free and overall survival curves to 1 year of age representing data from seventeen *Hesx1^Cre/+^;Ctnnb1^l^°^x(ex3)/+^* mice alongside the correlation between time to tumor identification and time between identification of tumor and death for twelve animals. Pearson correlation coefficient and one tailed significance analysis.

MRI and µ‐CT imaging performed at 8 weeks of age revealed enlargement of the pituitary of *Hesx1^Cre/+^;Ctnnb1^lox(ex3)/+^* mice relative to wildtype control mice (Figure 1A), consistent with the previously observed prenatal pituitary hyperplasia 11. Serial T_2_‐weighted MRI performed every 2 weeks revealed a relatively stable phase with no additional changes detectable, followed by lesion enlargement forming a solid tumor with associated cystic fluid accumulation (hyperintense relative to midbrain above; Figure 1B) and in most cases hemorrhage (relatively hypointense).

Tumor progression, defined as a change in imaging phenotype from the stable state, was identified in thirteen *Hesx1^Cre/+^;Ctnnb1^lox(ex3)/+^* mice prior to 1 year of age, at a median age of 17.7 weeks (range 8.3–35.3 weeks, *n* = 17) (Figure 1C). Median overall survival in the cohort was found to be 22.6 weeks (range 10.1–41.0 weeks). It was noted that tumors that presented later appeared to progress more slowly, and indeed the age at which progression was identified correlated significantly with the time from progression to sacrifice (Pearson *r* = 0.85, *P* = 0.0002).

### Multiparametric MRI reveals radiological features similar to human ACP, which correlate with μ‐CT and histology

**Figure 2:**
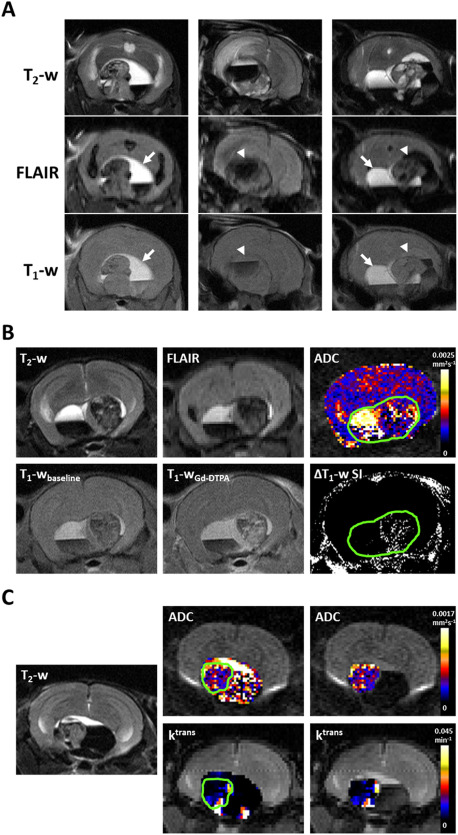
**A.** T_2_‐weighted (T_2_‐w), fluid attenuated inversion recovery (FLAIR) and T_1_‐weighted (T_1_‐w) MRI images from three *Hesx1^Cre/+^;Ctnnb1^l^°^x(ex3)/+^* mice demonstrating different cyst imaging presentation. Arrows denote cystic fluid that did not attenuate on FLAIR and was hyperintense on T_1_‐weighted MRI; arrowheads denote cystic fluid that attenuated on FLAIR and was isointense on T_1_‐weighted MRI. The example on the right shows both cyst phenotypes in the same tumor. **B. Upper panel**: T_2_‐weighted and FLAIR images, and a parametric map of apparent diffusion coefficient (ADC) acquired from a 1mm thick axial slice through a tumor ‐bearing *Hesx1^Cre/+^;Ctnnb1^l^°^x(ex3)/+^* mouse head. **Lower panel**: Matched T_1_‐weighted images acquired at baseline and 1 minute after injection of 0.1 mmol/kg Gd‐DTPA and a subtraction map clearly showing areas of signal enhancement. Green ROI denotes lesion volume. **C.** Parametric maps of ADC and transfer coefficient *K* ^trans^ in the entire lesion and the solid component of a *Hesx1^Cre/+^;Ctnnb1^l^°^x(ex3)/+^* tumor alongside a T_2_‐weighted anatomical image of the matched 1 mm slice. Green ROI denotes solid component of the lesion. Note the heterogeneous signal enhancement in the solid tumor components following contrast administration and high ADC in cystic areas in B and C.

Multiparametric MRI was used to assess the phenotype of progressing *Hesx1^Cre/+^;Ctnnb1^lox(ex3)/+^* tumors. Considerable heterogeneity of cystic components, identified by high T_2_‐weighted signal intensity, was noted both within and between tumors. In a subset of tumors (*n* = 5), these cysts remained hyperintense on FLAIR images, in which the signal from cerebrospinal fluid (CSF) and other motile fluid compartments would usually be suppressed, and were also relatively hyperintense on T_1_‐weighted images (Figure 2A left panel, arrow). In another subset (*n* = 5) cystic fluid attenuated to the same degree as ventricular CSF on FLAIR and was isointense to the midbrain on T_1_‐weighted images (Figure 2A, center panel, arrowhead). Both of these cystic phenotypes were also identified within individual lesions (*n* = 3, Figure 2A right panel) and were consistent with the imaging features of the cysts observed in ACP patients 10.

Quantitative functional MRI incorporating native and contrast‐enhanced parameters was also performed at study end. Parametric ADC maps demonstrated that whilst the solid tumor component appeared to have a similar ADC to the rest of the brain (Figure 2B), there was substantial heterogeneity in the tumors corresponding to the different components of the lesions (Figure 2B,C). Quantification of ADC in eight end‐stage tumors that consisted of all three tumor components showed that ADC was lowest in the solid component (635 ± 29 × 10^−6^ mm^2^ s^−1^), was significantly higher in the hemorrhagic component (856 ± 79 × 10^−6^ mm^2^ s^−1^, *P* = 0.02) and was higher still in the cystic regions (1953 ± 73 × 10^−6^ mm^2^ s^−1^, *P* < 0.0001 vs solid and hemorrhagic).

The uptake and distribution of Gd‐DTPA contrast agent in the tumors was assessed using two techniques. T_1_‐weighted images acquired prior to and following contrast agent administration provided qualitative data that showed heterogeneous signal enhancement in the solid tumor component but no response in the cystic or hemorrhagic components (Figure 2B). Quantitative maps of the transfer coefficient *K* ^trans^ (Figure 2C), which here represents a compound biomarker of perfusion and permeability, demonstrated heterogeneous distribution in the solid tumor component.

**Figure 3:**
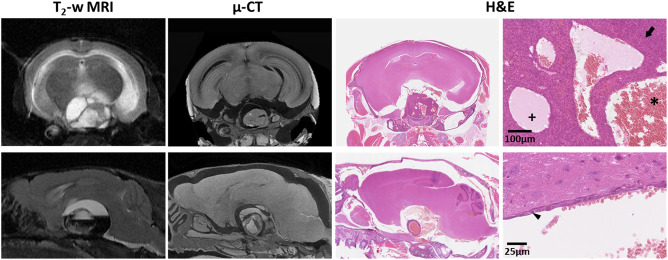
*In vivo* T_2_‐weighted MRI, *ex vivo* micro (µ)‐CT and H&E stained sections from two tumor‐bearing *Hesx1^Cre/+^;Ctnnb1^l^°^x(ex3)/+^* mouse heads. Snapshots of H&E staining were acquired at ×100 (upper panel) and ×400 (lower panel) magnification. Arrow denotes densely cellular solid tumor. Small cysts contained proteinaceous fluid (+) and/or red blood cells (*). Arrowhead indicates the simple epithelial layer that made up the wall of a large cyst. MRI slice thickness 1000 µm, μ‐CT slice thickness ≈ 9 µm, tissue sections 5 µm.

*Ex vivo* µ‐CT images of intact tumor‐bearing *Hesx1^Cre/+^;Ctnnb1^lox(ex3)/+^* mouse heads, which had been imaged *in vivo* by MRI prior to necropsy, provided higher resolution 3D visualization of the tumors *in situ*, confirming the complex architecture observed on MRI in addition to more detailed evaluation of microcystic components within the solid tumor (Figure 3 and Supporting Information Videos S1 and S2). No invasion of the brain parenchyma by the tumor was evident, and whilst smaller cysts within the solid tumor mass were preserved, the integrity of the larger cysts was in some cases difficult to assess because tissue shrinkage during fixation separated the tumor from the brain. H&E stained sections of the decalcified heads showed clinically relevant histological features observed by MRI and µ‐CT; for example densely cellular areas of solid tumor, cysts containing proteinaceous fluid and/or red blood cells, and cyst walls made up of a simple epithelial layer (Figure 3).

### Micro‐CT imaging of patient‐derived ACP xenografts reveals the 3D tumor structure and histological features of human ACP

**Figure 4:**
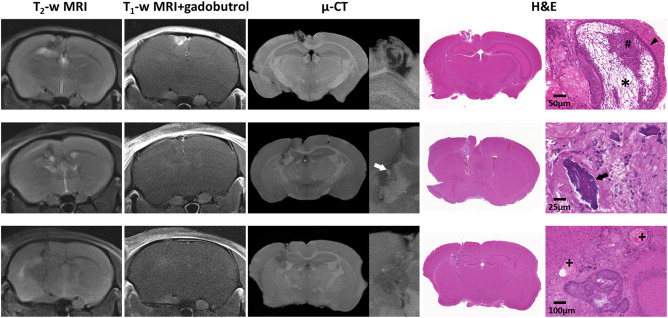
*In vivo* T_2_‐weighted and gadobutrol‐enhanced T_1_‐weighted MRI acquired immediately prior to necropsy, *ex vivo* micro (µ)‐CT and H&E stained sections from three mice bearing cerebrally implanted patient‐derived ACP xenografts. Histological features from the patient's tumor were maintained; cell clusters (#) palisading epithelium (arrowhead), stellate reticulum (*), calcification (arrows) and wet keratin (+). MRI slice thickness 700 µm, μ‐CT slice thickness ≈ 4–6 µm, tissue sections 3 µm.

MRI performed between 124 and 130 days after implantation of uncultured ACP tumor tissue into the cerebral hemisphere of nude mice demonstrated successful engraftment, with tumors appearing heterogeneous on both T_2_‐weighted and post‐contrast T_1_‐weighted images (Figure 4). *Ex vivo* µ‐CT of tumor‐bearing brains revealed heterogeneous tumors displaying imaging features that corresponded to histological features commonly observed in human ACP, such as palisading epithelium, epithelial whorls, stellate reticulum, wet keratin and calcification (Figure 4 and Supporting Information Videos S3–S5). These features were observed to varying degrees in each of the xenografts.

## DISCUSSION

The relevance of preclinical tumor models to human cancer has been a topic of debate for many years. Transgenic models, in which expression or knockout of a specific gene in the native tissue of origin leads to spontaneously arising tumors, orthotopic xenografts, where cancer cells are implanted in the organ from which they originated, and patient‐derived xenografts (PDXs), in which human tumor tissue is implanted directly from surgical or biopsy samples, are increasingly being exploited. The use of these models for preclinical cancer research must be underpinned by case‐specific evidence for each model establishing that tumor development, progression and radiology recapitulates the human disease. In this study we have used longitudinal *in vivo* MRI and high resolution *ex vivo* μ‐CT to compare and contrast the growth patterns and radiological features of tumors arising in *Hesx1^Cre/+^;Ctnnb1^lox(ex3)/+^* transgenic mice and in cerebrally implanted patient‐derived ACP xenografts.

MRI was first performed when *Hesx1^Cre/+^;Ctnnb1^lox(ex3)/+^* mice were approximately 8 weeks of age, where expansion and increased heterogeneity of the pituitary was observed in comparison with wildtype control mice. Due to the size of the mouse pituitary (∼1 mm × 1 mm × 3 mm) and the resolution achievable with *in vivo* MRI, it was not possible to detect the microscopic structural changes previously described, which were observed *in utero* as early as 9.5 days post coitum 11. Micro‐CT provided a method for high resolution *in situ* imaging, achieving resolution of ∼9µm^3^, and identified altered architecture of the anterior pituitary and also the posterior lobe of the gland. The high radiation exposure required to acquire images at this resolution and tissue contrast prohibits the use of this protocol *in vivo* and thus for longitudinal studies.

Anatomical T_2_‐weighted MRI provides a non‐invasive, non‐ionizing method of monitoring tumor growth over extended periods of time. In addition, advanced MRI techniques provide a means of defining non‐invasive quantitative biomarkers to inform on biologically relevant structure‐function relationships in tumors 5. Serial imaging of *Hesx1^Cre/+^;Ctnnb1^lox(ex3)/+^* mice revealed that, whilst the pituitary was enlarged from early postnatal life, there was indistinguishable growth during a stable period prior to progression, in some cases rapid, involving solid tumor growth, cystic expansion and other MRI detectable changes. Progressing tumors were identified from 8.3 to 35.3 weeks old (median 17.7 weeks), with 4/17 animals not demonstrating any imaging changes before 1 year of age. Overall survival, therefore, also differed greatly across the cohort with median survival being 22.6 weeks (4/17 animals alive beyond 1 year of age), considerably longer than the previously reported median survival of 11 weeks, with all animals dying before 6 months of age 11. These differences are likely the consequence of variations in the genetic background since the original study was performed. The time over which tumors progressed was also variable but we show that the age at which tumor progression was identified positively correlated with the time from progression to sacrifice; tumors that took longer to progress from the steady state, also progressed slower once expansion was identified. The molecular mechanisms underpinning this pattern of tumor evolution require further elucidation.

The formation of cysts is a hallmark in the presentation of ACP in children 19 and was observed during necropsy in the *Hesx1^Cre/+^;Ctnnb1^lox(ex3)/+^* model 11. Here, we show that the proportion of cystic component varies between tumors, ranging from microcysts within a largely solid lesion, to cysts making up approximately 70% of the abnormality. Most interestingly, in approximately half of the tumors analyzed some or all of these cysts appeared hyperintense on FLAIR images and T_1_‐weighted images, as is often observed in human ACP 10. This is thought to be caused by the presence of high levels of protein, cholesterol and blood breakdown products in the cystic fluid 1, 10, 12, contributing to its description as “engine oil”. Diffusion‐weighed MRI showed that the ADC of the cystic regions was significantly higher than the other regions of the tumors, demonstrating their fluid‐filled nature, and ADC values were indistinguishable between cysts that attenuated on FLAIR images and those that remained hyperintense. Diffusion‐weighted MRI has been investigated, alongside MR spectroscopy, for the evaluation of ACP in the clinic 25. Calcification of cyst walls, which is detectable by CT in patients and is integral in the differential diagnosis of ACP 29, was not observed in *Hesx1^Cre/+^;Ctnnb1^lox(ex3)/+^* tumors by *ex vivo* µ‐CT, which may be related to the relatively short evolution time of mouse tumors compared to human tumors.

The solid component of the tumors, which in some cases did not enlarge far beyond the original size of the abnormal pituitary, were in themselves heterogeneous with small areas of hypointensity on T_2_‐weighted MRI and evidence of microcysts in some cases. These smaller cysts were also apparent on μ‐CT and histologically, consistent with some human ACPs 22. As expected, these regions displayed the most restricted diffusion of water molecules, demonstrating ADC values equivalent to those measured in the normal mouse striatum and intracranially implanted murine tumors 5, 23. Gadolinium contrast agent extravasation occurred heterogeneously in the solid portion of the tumor only, recapitulating the pattern of enhancement in patient tumors 10. In patients, some rim enhancement in the cysts occurs; this was not observed preclinically but may be a feature of the relatively smaller tumors and relatively lower resolution. Imaging and pathological evidence showed that, unlike in patients, the tumors that develop in *Hesx1^Cre/+^;Ctnnb1^lox(ex3)/+^* mice do not invade into the brain, which is likely to be as a result of differences in the hypothalamo‐pituitary axis anatomy between mice and humans 18.

The majority of mice (11/13) presented with regions of hypointensity on T_2_‐weighted images, which were either hypointense or isointense with the brain parenchyma on T_1_‐weighted images, and thus consistent with the presence of paramagnetic species, such as deoxyhemoglobin, ferritin and hemosiderin, from the blood and degradation of erythrocytes associated with hemorrhage. The size of these regions varied in size up to approximately 70% of the lesion volume in the most extreme case. ADC in these regions was significantly higher than in the solid component of the tumors but was approximately 2.4‐fold lower than the fluid in the cysts, suggesting that there are substantial barriers to the diffusion of water molecules in these regions. Hemorrhage was also identifiable on µ‐CT images and H&E staining. The combination of cystic volume and large hemorrhagic regions, leading to raised intracranial pressure, was likely the predominant cause of morbidity in the mouse cohort.

Patient‐derived ACP xenografts also showed MRI features similar to human ACP, consistent with the initial description of the model 26. In addition, µ‐CT gave further 3D insight into the architecture of these tumors, and highlighted several features of human ACP, including calcification and specific histological structures such as stellate reticulum, wet keratin and palisading epithelium (Figure 4).

As druggable targets are increasingly identified in human and mouse studies [reviewed in 3] it will be important to develop appropriate preclinical strategies to test novel therapeutic agents. The two models described here are complementary and recapitulate many of the radiological and pathological aspects of human ACP. We anticipate that the use of these murine ACP models will improve the accuracy of preclinical data and accelerate the development of urgently required targeted therapies for these devastating human tumors.

## Supporting information

Additional Supporting Information may be found in the online version of this article at the publisher's web‐site:

**Video S1.** Micro‐CT volume rendering (isotropic voxel size=9.1 µm) demonstrating virtual dissection of the tumor that arose in the *Hesx1^Cre/+^;Ctnnb1^lox(ex3)/+^* mouse shown in the upper panel of Figure 3. Complex areas of cystic and solid tumor can be identified, along with compression of the surrounding brain parenchyma.

**Video S2.** Micro‐CT volume rendering (isotropic voxel size=8.7 µm) demonstrating virtual dissection of the tumor that arose in the *Hesx1^Cre/+^;Ctnnb1* ^*lox(ex3)/+*^ mouse shown in the lower panel of Figure 3. Complex areas of cystic and solid tumor can be identified, along with compression of the surrounding brain parenchyma.

**Video S3.** Micro‐CT volume rendering (isotropic voxel size=3.7 µm) demonstrating virtual dissection of the cerebrally implanted patient‐derived ACP xenograft shown in the upper panel of Figure 4. High spatial resolution and differential uptake of iodine‐based contrast enables visualization of a cluster within an area of stellate reticulum.

**Video S4.** Micro‐CT volume rendering (isotropic voxel size=6.1 µm) demonstrating virtual dissection of the cerebrally‐implanted patient‐derived ACP xenograft shown in the middle panel of Figure 4. A radio‐dense focus can be seen within the xenograft; this corresponds to an area of calcification on histological examination.

**Video S5.** Micro‐CT volume rendering (isotropic voxel size=3.8 µm) demonstrating virtual dissection of the cerebrally‐implanted patient‐derived ACP xenograft shown in the lower panel of Figure 4. Islands of wet keratin adjacent to solid tumor (confirmed on H&E, Figure 4) give the xenograft a heterogeneous appearance on micro‐CT examination.
